# Characterization of infertile couples and outcomes of their first *in vitro* fertilization cycle at public fertility centers in Northern and Central Portugal: a five-year cohort study

**DOI:** 10.5935/1518-0557.20250049

**Published:** 2025

**Authors:** Emídio Vale-Fernandes, Ricardo Sousa-Santos, Renato Silva-Martins, Fátima Silva, Filipa Brás, Andreia Leitão-Marques, Sandra Silva-Soares, Marta Osório

**Affiliations:** 1 ICBAS - School of Medicine and Biomedical Sciences, UMIB - Unit for Multidisciplinary Research in Biomedicine, University of Porto, Porto, Portugal; 2 Centro de Procriação Medicamente Assistida / Banco Público de Gâmetas, Centro Materno-Infantil do Norte Dr. Albino Aroso (CMIN), Centro Hospitalar Universitário de Santo António (CHUdSA), Unidade Local de Saúde de Santo António (ULSSA), Porto, Portugal; 3 ITR - Laboratory for Integrative and Translational Research in Population Health, University of Porto, Porto, Portugal; 4 Hospital Senhora da Oliveira (HSO), Unidade Local de Saúde do Alto Ave (ULSAV), Guimarães, Portugal; 5 Cintesis - Centro de Investigação em Tecnologias e Serviços de Saúde, Faculdade de Medicina da Universidade do Porto, Porto, Portugal; 6 Centro de Investigação em Ciências da Saúde, Faculdade de Ciências da Saúde da Universidade da Beira Interior, Covilhã, Portugal; 7 Centro Hospitalar Universitário Cova da Beira (CHUCB), Unidade Local de Saúde da Cova da Beira (ULSCB), Covilhã, Portugal; 8 Centro Hospitalar de Vila Nova de Gaia/Espinho (CHVNG/E), Unidade Local de Saúde de Gaia/Espinho (ULSGE), Vila Nova de Gaia, Portugal; 9 Centro Hospitalar e Universitário de Coimbra (CHUC), Unidade Local de Saúde de Coimbra (ULSC), Coimbra, Portugal; 10 Centro Hospitalar Universitário de São João (CHUSJ), Unidade Local de Saúde de São João (ULSSJ), Porto, Portugal

**Keywords:** infertility, assisted reproductive techniques, live birth rate, cumulative live birth rate

## Abstract

**Objective:**

Infertility is a major global health issue, with assisted reproductive technologies (ART) commonly employed as a solution. Although numerous factors related to couple characteristics and ART cycles are implicated in pregnancy outcomes, data on infertile couples in Portugal are limited. The Portuguese Northern and Central Regions Infertility Study 2014-2018 (NORCE-INF 14-18) aimed to evaluate how couple characteristics and *in vitro* fertilization (IVF) cycle parameters affect the success of the first IVF treatment, specifically through cumulative live birth rate (CLBR).

**Methods:**

This multicenter, observational, longitudinal, retrospective cohort study gathered data from six public fertility centers in Northern and Central Portugal, involving couples undergoing their first IVF treatment from January 2014 to December 2018, along with subsequent frozen embryo transfers (FET).

**Results:**

The analysis included 5,250 couples, with mean female and male ages of 34.3 and 36.1 years, respectively. The average duration of infertility was 45.88 months, predominantly resulting from male factor infertility. Among 3,767 cycles with fresh embryo transfers, the live birth rate was 29.5%. Additionally, the study found a CLBR of 25.5%, leading to 1,601 live births. Logistic regression identified female age as a key predictor of ART success, showing an inverse relationship with pregnancy rates.

**Conclusions:**

The NORCE-INF 14-18 study represents the largest analysis of ART outcomes in Portugal, revealing that approximately 26% of couples achieved live births after their first IVF cycle, emphasizing the importance of demographic factors in treatment success.

## INTRODUCTION

Infertility is a worldwide health care issue, affecting approximately one in every six couples, with important psychological, economic, demographic and medical implications (Feichtinger *et al*., 2016; Michau *et al*., 2019). The causes of infertility include male and female-related factors, such as: ovulatory disorders (age or non-age related), Fallopian tube abnormalities, endometriosis, uterine abnormalities and cervical factor. A considerable proportion of infertile couples remain without a diagnosis (Vaegter *et al*., 2017). Some causes of infertility are easily identifiable, such as: azoospermia, longstanding oligo/amenorrhea, and bilateral tubal obstruction. However, the situation is less clear in some couples. The uncertain relationship between a test anomaly and the actual cause of infertility makes it difficult to estimate the relative frequency of its causes (Hull *et al*., 1985; Vaegter *et al*., 2017).

The assisted reproductive techniques (ART) are the solution for many causes of infertility and several factors can affect the pregnancy rate in a couple. According to the literature there are some pretreatment patients variables that can predict the success of infertility treatment such as: female age, body mass index (BMI), ethnicity, ovarian reserve markers [anti-Müllerian hormone (AMH) and antral follicle count (AFC)], tobacco use, cause of infertility and duration of infertility (McQueen *et al*., 2015; Dhillon *et al*., 2016; Luke, 2017). For instance, pregnancy rates decrease significantly with women’s aging whereas anovulatory infertility seems to be associated with good outcomes (Vaegter *et al*., 2017).

Regarding BMI, and since the incidence of obesity is continually rising, an increasing number of overweight and obese women are seeking fertility treatments. The global impact of obesity on ART is conflicting. Some studies report a decreased embryo quality in obese women, with decreased live birth rates and increased miscarriage rates. However, the majority of studies did not show a significant effect regarding this issue (Ozekinci *et al*., 2015; Wang *et al*., 2016; Luke, 2017). It has been shown that tobacco use is associated with reproductive impairments in a dose-dependent manner, namely, diminished ovarian reserve and/or response, reduced uterine receptivity, and adverse pregnancy outcomes (Dechanet *et al*., 2011; Zhang *et al*., 2018). Concerning the male partner, some studies have provided no evidence that advancing age, elevated BMI, and abnormal sperm parameters significantly affect ART success or obstetric outcomes (Wang *et al*. 2016; Vaegter *et al*., 2017; Capelouto *et al*., 2018).

There is a lack of data regarding the infertile couple’s characteristics and ART outcomes in the Portuguese population, which motivated the conduction of this multicenter study.

The primary objective of the NORCE-INF 14-18 study, conducted in the Northern and Central Regions, was to evaluate the impact of couple characteristics and *in vitro* fertilization (IVF) cycle parameters on the success rates of the initial IVF treatment among infertile couples seeking assistance at public infertility centers between the years 2014 and 2018.

The secondary objectives of this study were to characterize infertile couples initiating treatment at public infertility centers who are undergoing their first IVF cycle, as well as to analyze the outcomes and complications associated with this initial IVF cycle. Furthermore, the study aimed to assess the obstetric outcomes associated with these couples.

## MATERIAL AND METHODS

An observational, longitudinal, retrospective cohort study was conducted utilizing data from infertile couples undergoing their first IVF treatment at six public fertility centers in Northern and Central Portugal: Centro Materno- Infantil do Norte (CMIN) - Centro Hospitalar Universitário de Santo António (CHUdSA), Hospital Senhora da Oliveira (HSO) - Guimarães, Centro Hospitalar Universitário de São João (CHUSJ), Centro Hospitalar de Vila Nova de Gaia/Espinho (CHVNG/E), Centro Hospitalar e Universitário de Coimbra (CHUC), and Centro Hospitalar Universitário Cova da Beira (CHUCB), covering the period from January 2014 to December 2018.

Inclusion criteria consisted of infertile couples who underwent their first IVF cycle at these ART centers, with oocyte retrieval performed between January 2014 and December 2018. Exclusion criteria included canceled treatments prior to oocyte retrieval; cycles utilizing donated gametes; cycles involving vitrified oocytes; cycles conducted for preimplantation genetic testing and embryo selection; cycles with missing or erroneous mandatory data; and fertility preservation cycles.

The primary data source for this study consisted of the local databases routinely employed in each participating center for ongoing treatments. Data extraction was fully anonymized for statistical analysis, employing a unique random identifier for each cycle or couple.

This study adhered to applicable legal and regulatory requirements, as well as established research practices, and received approval from the Independent Ethics Committee (IEC) of each participating center. Due to the non-interventional nature of the study, the reliance on data collected in accordance with routine practice, the anonymized analysis, and the presentation of results in aggregate form (with no identifiable patient information), a waiver for obtaining Informed Consent Forms was granted by all IECs.

### Endpoint description and definition

The objective of this study is to analyze the influence of couple and IVF cycle characteristics on the success of the first IVF treatment among infertile couples attending public infertility centers between 2014 and 2018.

To achieve this primary objective, the analysis focuses on the effects of factors such as age, tobacco use, BMI, duration of infertility, the number of oocytes retrieved, and the day of embryo transfer on the success of the initial IVF treatment. Success is defined in terms of the cumulative live birth rate (CLBR) among infertile couples.

In this context, live birth is characterized as any birth event resulting in at least one live-born infant weighing more than 500 grams and gestating for more than 22 weeks.

### Cumulative live birth rate (CLBR)

The primary endpoint of this study is to measure the CLBR, adopting a conservative approach, where each oocyte retrieval cycle represents a single event, and outcomes from any type of embryo transfer associated with that retrieval are considered [fresh embryo transfer and frozen embryo transfer (FET)]. This gives us the global probability of success for a couple reaching oocyte pickup. The denominator does not exclude 0 oocytes and/or 0 transfers:


$$\frac{\text{ Sum of all couples with at least one live birth, either from fresh or frozen transfer }}{\text{ first IVF cycles reaching oocyte pickup }}$$

### Live birth rate (LBR)

The live birth rate (LBR) per transfer and per transferred embryo was also calculated as follows:


$$\frac{\text{ Sum of live births from fresh or frozen transfer }}{\text{ Number of cycles with transferred embryos }};$$

$$\frac{\text{ Sum of live biths from fresh or frozen transfer }}{\text{ Number of embryos transferred }}$$

### Clinical pregnancy rate (CPR)

Clinical pregnancy was defined as a clinically identifiable gestational sac (with or without embryo). The clinical pregnancy rate (CPR) corresponds to the number of cycles with clinical pregnancy over the total number of cycles.

### Cumulative clinical pregnancy rate (CCPR)


$$\frac{\text{Sum of all cycles with clinical pregnancies from fresh transfer or FET}}{\text{first IVF cycles reaching oocyte pickup}}$$

### Miscarriage rate

Miscarriage was defined as fetal loss before 22 weeks (154 days) or fetal weight less than 500 grams. Miscarriage was divided into early (≤12 completed weeks from day of oocyte pickup plus 14 days) and late (≥13 weeks).

Miscarriage rate is the proportion of women with a spontaneous fetal loss before 22 weeks among those women with a clinical intrauterine gestation.

Calculation was done by:


$$\frac{\text{Number of non viable pregnancies}}{\text{Sum of all clinical pregnancies from fresh transfer or FET}}$$

Miscarriage rate will be also decomposed by fresh/frozen transfers:


$$\frac{\text{Number of non viable pregnancies after fresh transfer}}{\text{Sum of all clinical pregnancies after fresh transfer}};\frac{\text{Number of non viable pregnancies after FET}}{\text{Sum of all clinical pregnancies after FET}}$$

### Multiple pregnancy rate

Multiple pregnancies rate was calculated dividing clinically identifiable multiple embryos per clinical pregnancy, transfer and oocyte pickup:


$$\frac{\text{ Number of clinically identifiable multiple pregnancies. }}{\text{Number of clinical pregnancies Number of clinically identifiable multiple pregnancies.}};\frac{\text{Number of transfers Number of clinically identifiable multiple pregnancies }}{\text{Number of pickups}}$$

Multiple birth was defined as the delivery of two or more newborns at or after 22 weeks (154 days) of gestation:


$$\frac{\text{Number of multiple births}}{\text{Number of clinical pregnancies'}};\frac{\text{Number of multiple births }}{\text{Number of transfers }};\frac{\text{Number of multiple births }}{\text{Number of oocyte pickups }}$$

### Prematurity

Gestational age is given in weeks (w). Cut Offs will be *preterm* (<37w), *very preterm* (<32w) and *extremely preterm* (<28w).

### Elective single embryo transfer (eSET)

eSET is defined as a fertility treatment strategy wherein a single viable embryo is transferred from a cohort of multiple embryos, where at least one additional embryo of similar or superior quality would be suitable for subsequent cryopreservation and potential transfer in a subsequent cycle.

The pregnancy rate after eSET was calculated by the ratio of the number of cycles with clinical pregnancy after eSET over the total number of cycles where eSET was performed.

The pregnancy rate after 2 embryos transfer was calculated by the ratio of the number of cycles with clinical pregnancy after transfer of 2 embryos over the total number of cycles where transfer of 2 embryos was performed.

### Freeze all rate

The proportion of initial IVF cycles resulting in transferable embryos that were not subjected to a fresh transfer was assessed.

### Statistical considerations

Data analysis was conducted using IBM SPSS version 26. For the comparison of parametric data, Student’s t-test and analysis of variance (ANOVA) were employed, while the Kruskal-Wallis test was utilized for the analysis of non-parametric data. Distributions and variances were assessed to determine the appropriate statistical tests. Pearson’s Chi-Square test and Fisher’s exact test were applied for the analysis of categorical data. Additionally, binary logistic regression was performed using the Enter method for model building.

## RESULTS

### Population characterization

A total of 5,250 infertile couples met the inclusion criteria (Table 1). The mean age of female participants was 34.3 years, which exhibited an increase of 5.4 months from 2014 to 2018 (34.1 *vs*. 34.6; *p*=0.001). Table 2 illustrates a decline in the proportion of heterosexual couples with a female partner under 31 years of age, alongside an increase in couples with a female partner over 38 years. The majority of couples identified as Caucasian (female: 97.7%; male: 98.1%) and non-smokers (non-smokers: female 79.1%, male 62.1%; smokers: female 15.5%, male 29.4%). The mean BMI for females was 23.7 kg/m^2^, with 33.8% categorized as overweight or obese. The mean BMI for males was 26.08 kg/m^2^, with 61.5% classified as overweight or obese. The average duration of infertility was 45.9 months (range: 0-180 months), translating to a median duration of 3.8 years (0-15 years). Primary infertility was reported in 93.3% of cases, with male factor infertility identified as the most prevalent cause, occurring in 31.3% of cases in isolation and in 51.9% of cases when female-related factors were included.

**Table 1:** Overall demographics and infertility description of the study cohort (n=5250).

Demographics	Female	Male
Age (years)	34.31 (19-40)	36.1 (20-59)
BMI (Kg/m^2^) Underweight Normal weight Overweight Obese Missing/wrong data	23.74 (15-41) 195 (3.7%) 3275 (62.4) 1258 (24%) 516 (9.8%) 6 (0.1%)	26.08 (17-48) 31 (0.6%) 1747 (33.3%) 2499 (47.6%) 728 (13.9%) 245 (4.7%)
Ethnicity White Non-white	5128 (97.7%) 122 (2.3%)	5151 (98.1%) 99 (1.9%)
Tobacco use Present Past None	816 (15.5%) 283 (5.4%) 4151 (79.1%)	1543 (29.4%) 445 (8.4%) 3262 (62.1%)
**Infertility description**		
Duration in months/years	45.88 (0-180)/ 3.8 (0-15)	
Primary Secondary (couple with children)	4899 (93.3%) 351 (6.7%)	
Etiology Male factor Female factor(s) Female and male factors Endometriosis Idiopathic Others Missing data	1643 (31.3%) 1466 (27.9%) 1084 (20.6%) 284 (5.4%) 722 (13.8%) 35 (0.7%) 16 (0.3%)	

**Table 2:** Female Age analysis per year of ART treatment [*p=0.012* (*Chi-Square*)].

% within treatment year							
		**Year of treatment**					**Total** **(2014-2018)**
		**2014**	**2015**	**2016**	**2017**	**2018**	
Female age	<31	17.9%	17.6%	15.3%	14.3%	14.8%	15.9%
	31-34	32.7%	31.8%	32.5%	29.7%	29.8%	31.3%
	35-37	26.2%	30.4%	29.7%	29.5%	29.4%	29.0%
	38-40	23.2%	20.2%	22.5%	26.6%	26.0%	23.8%

### IVF cycle characteristics

The majority of ovarian stimulation protocols implemented in the analyzed sample were of the short type utilizing gonadotropin-releasing hormone (GnRH) antagonists, accounting for 81.2% of cases. Among these, nearly 68% employed recombinant drugs, 15% utilized highly purified human menotropin (HP-HMG), and approximately 17% utilized a combination of both formulations. The mean dosage of total medication administered was 2,009 International Units (IU), with a minimum of 75 IU and a maximum of 9,750 IU. In nearly 45% of IVF cycles, the number of oocytes retrieved during the pickup ranged from 4 to 9, while 12% of patients were classified as high responders, yielding more than 15 oocytes. There were 97 instances (1.8%) where no viable oocytes were retrieved. The mean number of oocytes retrieved was 8.6, with an average of 7.2 fertilized or injected oocytes and a mean of 3.8 embryos obtained. Detailed cycle characteristics are summarized in Tables 3 and 4.

**Table 3:** IVF Cycle Characteristics.

Type of Ovarian Stimulation		Drug Regimen for Ovarian Stimulation	
Short Protocol Long Protocol Other Non-Pharmacological	4401 (83.8%) 825 (15.7%) 10 (0.2%) 14 (0.5%)	GnRH Agonist GnRH Antagonist *Missing* Other	934 (17.8%) 4264 (81.2%) 17 (0.3%) 35 (0.7%)
**Drug Type**		**Total dose of drug used**	
Recombinant Urinary Both *Missing*	3547 (67.6%) 771 (14.7%) 881 (16.8%) 51 (1%)	Overall Mean Recombinant Recombinant + Urinary Urinary	2009 (75-9750) 1797 (75-6825) 2666 (600-9750) 2289 (150-7200)
**Origin of Spermatozoa**		**Type of ART treatment**	
Ejaculate *Fresh* *Cryopreserved* *Electrostimulation* Non-Ejaculate (biopsy) *Missing*	4851 (92.4%) 4752 (90.5%) 93 (1.8%) 6 (0.1%) 183 (3.5%) 224 (4.1%)	Classical IVF Mix (IVF + ICSI) ICSI *Missing*	2846 (54.2%) 129 (2.5%) 2273 (43.3%) 2

**Table 4:** Oocyte pick up (OPU), oocyte fertilization and embryo data.

Number of Oocytes		Number of Oocytes at OPU	
None < 4 4 to 9 10 to 15 > 15 16 to 20 > 20	97 (1.8%) 912 (17.4%) 2338 (44.5%) 1274 (24.3%) 629 (12%) 389 (7.4%) 240 (4.6%)	Mean/median OPU (range)	8.6/7 (0-35)
		**Inseminated or Fertilized Oocytes**	
		Mean/median (range)	7.1/6 (0-30)
		**Obtained Embryos**	
		Mean/median (range)	3.8/3 (0-25)

**Figure 1:**
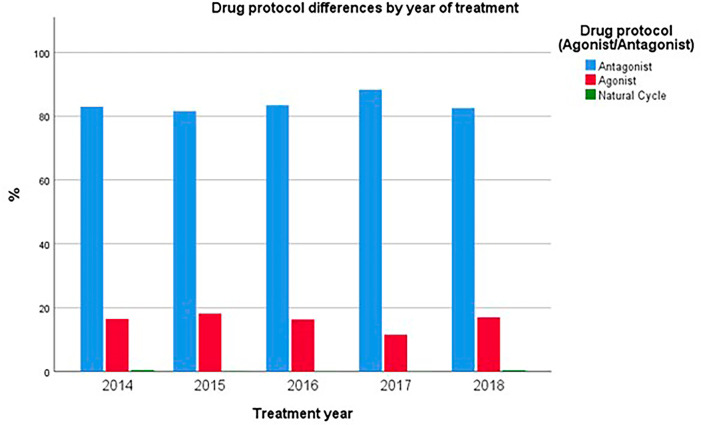
Drug protocol used by year of treatment.

The GnRH agonist protocol was employed in varying percentages of cycles, ranging from 18.2% in 2015 to 11.6% in 2017 (*p*=0.03), while natural cycles accounted for a negligible number (1-5 per year). The use of agonist protocols was least prevalent among couples with male factor infertility (10% of these cycles) and most frequent in couples where the female partner had a diagnosis of endometriosis (55% of these cycles; *p*<0.001) (Figure 1).

**Figure 2:**
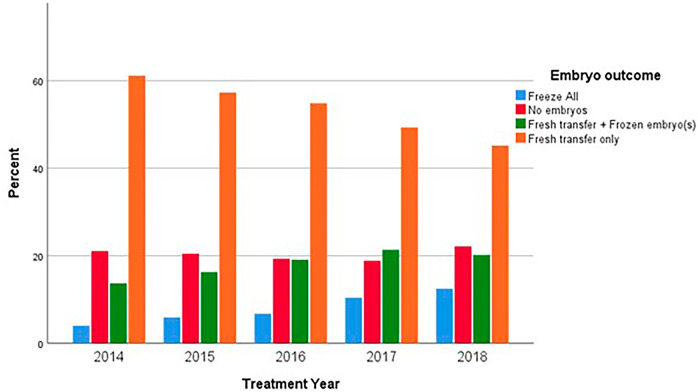
Type of embryo transfer over the studied period.

Throughout the cohort period, no statistically significant differences were observed in the number of oocytes retrieved. However, significant differences were noted in the number of embryos produced (higher in 2016, *p*=0.01), as well as in the number of embryos cryopreserved, which exhibited a growing trend and peaked in 2018 (*p*<0.001). The analysis also revealed significant differences in subsequent FET cycles *(p*<0.001). Figure 2 illustrates the observed trend towards an increase in “freeze-all” cycles and a decrease in fresh-only transfers, alongside a rise in combined freeze and fresh transfers (*p*<0.001).

**Figure 3:**
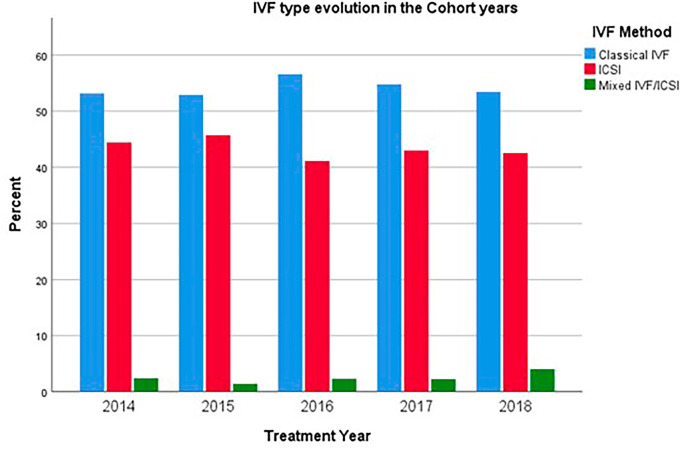
IVF type.

The usage patterns of intracytoplasmic sperm injection (ICSI), classical IVF, and mixed cycles exhibited significant changes over the years, specifically indicating a trend towards a reduction in the utilization of ICSI (*p*=0.013) (Figure 3).

### Fresh embryo transfer cycles

A total of 3,767 cycles involving fresh embryo transfer were recorded, comprising 2,239 double embryo transfers (DET), 1,515 single embryo transfers (SET), and 12 triple embryo transfers (TET). eSET was conducted in 429 cycles, representing 11% of all fresh transfers.

Table 5 delineates the distribution of SET, DET, and TET cycles, both with and without the inclusion of frozen embryos. The majority of embryo transfers (50.5%) occurred on day 3, followed by 24.8% on day 5. The distribution of cycles according to transfer day is also presented in Table 6.

**Table 5:** Single Embryo Transfers (SET), Double Embryo Transfers (DET), and Triple Embryo Transfers (TET) with and without additional frozen embryos.

**Number of cycles**	**SET with frozen (eSET)**	**SET without frozen**	**DET with frozen**	**DET without frozen**	**TET with frozen**	**TEF without frozen**
	429	1086	526	1713	1	11
	1515		2239		12	

**Table 6:** Number of cycles per transfer day. Single Embryo Transfers (SET), Double Embryo Transfers (DET), and Triple Embryo Transfers (TET).

Day of transfer	D2	753	SET	385
			DET	366
			TET	1
	D3	1906	SET	1025
			DET	1622
			TET	11
	D4	150	SET	39
			DET	111
	D5	936	SET	436
			DET	499
			TET	1
	D6	9	SET	15
			DET	7

Among the cohort, there were 1,483 couples (28.4%) who did not undergo a fresh embryo transfer. Of these, 1,067 cycles (20.3%) were attributed to the absence of transferable embryos, while 416 cycles were categorized as “freeze all” cycles. The mean number of oocytes retrieved in the “freeze all” cycles was 16.6, with a range of 1 to 35. These “freeze all” cycles constituted 10% of the 4,183 cycles with transferable embryos and represented 7.9% of the overall cycles.

In terms of complications, there were 22 reported cases (0.4%), which included 19 instances of moderate to severe ovarian hyperstimulation syndrome (OHSS) and three cases of pelvic inflammatory disease. The mean number of oocytes retrieved in the cases of OHSS was 21, with a range varying from 5 to 35 oocytes. Of the OHSS cases, “freeze all” was practiced in 13 instances.

In summary, the analysis encompassed 5,250 cycles, which subsequently resulted in 1,372 cryopreservation cycles.

#### bstetric Outcomes

During the cohort study period, a total of 1,355 clinical pregnancies were recorded in fresh cycles. Among these, 1,108 were classified as singleton pregnancies (81.8%), while 247 were multiple pregnancies (18.2%), which included three cases of triplets.

Miscarriage occurred in 230 clinical pregnancies (17%), encompassing losses in both the first and second trimesters. Additionally, there were 13 terminations of pregnancy (TOP), of which five were due to chromosomal abnormalities (two cases of trisomy 21, one case of trisomy 18, one case of triploidy, and one case of Turner syndrome). Three terminations were attributed to premature rupture of membranes prior to viability, and one was due to intestinal obstruction. The diagnoses or causes of the remaining four TOP cases were not documented. Among singleton pregnancies, there were four instances of intrauterine fetal death, and one twin in a pair experienced intrauterine fetal demise at 23 weeks of gestation, with the surviving twin being delivered at 30 weeks of gestation. Furthermore, the study recorded 16 ectopic pregnancies (1.37%) as well as one heterotopic pregnancy (0.085%) resulting from fresh embryo transfer cycles.

The mean gestational age at delivery for pregnancies resulting from fresh cycles was 37.5 weeks, with a median of 38 weeks and a range from 22 weeks to 43 weeks. Prematurity rates noted included 0.8% of infants born before 28 weeks (9 out of 1,110), 3% born between 28 and 31 weeks (33 out of 1,110), and 19.4% born between 32 and 37 weeks (216 out of 1,110). Data on gestational age were missing for six cases (6 out of 1,116).

The overall prematurity rate was calculated to be 23.2%, with rates of 12.5% for singleton pregnancies and 70.7% for multiple pregnancies. Specifically, prematurity rates were 2.4% for births occurring before 28 weeks, 7.8% for those between 28 and 31 weeks, and 60.5% for births between 32 and 36 weeks.

Regarding the mode of delivery in fresh cycles, 51.8% of deliveries were vaginal, and 48.2% were via cesarean section, with data missing for 3.7% of the deliveries.

#### Pregnancy rates

Pregnancy rates are detailed in Table 7. The CPR per transfer was 35.9%, encompassing a total of 1,355 cases, while the multiple pregnancy rate per clinical pregnancy was observed at 18.2%, amounting to 247 cases. The LBR per transfer in fresh cycles was calculated to be 29.5%, corresponding to 1,112 live births out of 3,767 transfers. This total includes various pregnancy outcomes, including singleton, multiple, and triplet pregnancies, resulting in an aggregate of 1,321 live newborns.

**Table 7:** Pregnancy Rates, Clinical Pregnancy Rates and Live Birth Rates.

Clinical Pregnancy Rate - CPR		Live Birth Rate - LBR	
/ *per IVF cycle*	1355 / 5250 (25.8%)	/ *per IVF Cycle*	1112/ 5250 (21.2%)
/ *per embryo transfer*	1355 / 3767 (35.9%)	*/ per embryo transfer*	1112 / 3767 (29.5%)
**Multiple Pregnancy rate**	247 / 1355 (18.2%)	**Live newborns/ per embryo transferred**	1321 / 6029 (21.9%)
**Multiple birth rate**	207 / 1112 (18.5%)	**Miscarriage Rate**	230 / 1355 (17%)
**Clinical Pregnancy Rate - eSET**	171/ 429 (39.9%)	**Clinical Pregnancy Rate - DET**	920 / 2239 (41.1%)

For eSET, the live birth rate was determined to be 35.4%, with a statistically significant variation based on the day of transfer. The LBR ranged from 23% for transfers on Day 3 (D3) to 47% for transfers on Day 5 (D5) (*p*=0.001; Table 8).

**Table 8:** Live birth on elective Single Embryo Transfers (eSET) according to the day of transfer [Fishers exact *p*=0.001].

			Day of fresh eSET transfer					Total
			2	3	4	5	6	
Live birth	No	Count	17	159	6	93	2	277
		% of Day of transfer	77.3%	72.3%	60.0%	53.1%	100.0%	64.6%
	Yes	Count	5	61	4	82	0	152
		% of Day of transfer	22.7%	27.7%	40.0%	46.9%	0%	35.4%
Total		Count	22	220	10	175	2	429
		% of Day of transfer	100%	100%	100%	100%	100%	100%

### Frozen embryo transfer (FET) cycles

Among the 5,250 cycles analyzed, 1,372 yielded at least one frozen embryo, and 1,246 FET cycles were subsequently identified through February 2020. The proportion of FET cycles increased over time, rising from 6.1% in 2014 to 27% of the total cohort in 2018.

The number of embryos transferred significantly decreased from 2014 to 2018, indicating a trend towards single embryo transfer (*p*<0.001). Furthermore, a shift in the timing of embryo transfer was observed, with Day 5 (D5) transfers increasing from 19% in 2014 to 52% in 2019, while Day 3 (D3) transfers decreased from 46% to 25% during the same period (*p*<0.001; Table 9).

**Table 9:** Number of transferred embryos per Frozen Embryo Transfers (FET) per year.

			Number of transferred embryos				Total
			0	1	2	3	
Year	2014	Count	13	23	40	0	76
		% within Year	17.1%	30.3%	52.6%	0.0%	100.0%
	2015	Count	22	58	68	0	148
		% within Year	14.9%	39.2%	45.9%	0.0%	100.0%
	2016	Count	20	76	133	1	230
		% within Year	8.7%	33.0%	57.8%	0.4%	100.0%
	2017	Count	22	120_b_	153	0	295
		% within Year	7.5%	40.7%	51.9%	0.0%	100.0%
	2018	Count	25	197_b_	115	0	337
		% within Year	7.4%	58.5%	34.1%	0.0%	100.0%
	2019	Count	14	94	52	0	160
		% within Year	8.8%	58.8%	32.5%	0.0%	100.0%
Total		Count	116	568	561	1	1246
		% within Time Period	9.3%	45.6%	45.0%	0.1%	100.0%

Within the 1,246 FET cycles, 116 (9.3%) did not result in a viable embryo for transfer post-thawing. The CPR per transfer was 30.2%; however, among these clinically confirmed pregnancies, the proportion of miscarriages was 29.9%, which included five late miscarriages. Additionally, there were two cases of medical termination of pregnancy due to trisomy 13 and trisomy 18, as well as five ectopic pregnancies.

More than one-fifth (20.6%) of couples with at least one frozen embryo achieved a successful pregnancy that culminated in labor, resulting in a live birth rate per transfer of 21.1%. The multiple pregnancy rate per clinical pregnancy was 14.8% with a multiple live birth rate of 16.1% (38 multiple births out of 236 live births, including one case of triplets).

Among the 1,246 cryo-cycles examined, 559 (44%) were classified as natural or modified natural cycles, while 672 (53.9%) were categorized as artificial cycles. Natural cycles exhibited a higher live birth rate and a lower miscarriage rate compared to artificial cycles (22.6% *vs*. 20.1% and 22.4% *vs*. 33.7%, respectively), though these differences were not statistically significant. The CPR between natural and artificial cycles were comparable (29.2% *vs*. 30.9%).

The number of embryos transferred per cycle and the timing of the embryo transfer were similar between the two groups. Regarding obstetric outcomes, the median gestational age at birth for singleton pregnancies was 38 weeks (ranging from a minimum of 24 weeks to a maximum of 41 weeks), while for multiple pregnancies, it was 36 weeks (ranging from 26 to 38 weeks) with statistical significance (*p*<0.001). The rate of prematurity was 13.9% for singletons and 53.8% for multiples.

The overall cesarean delivery rate among FET pregnancies was 58%. Data on the type of labor (spontaneous versus induced) are not available. Additionally, no neonatal outcome data were collected in this analysis.

### Cumulative results

Among all cases included in the study, 78.6% underwent at least one embryo transfer, whether fresh or frozen, with 66.2% receiving a single embryo transfer and 9% receiving two embryo transfers. Within this population, 71.8% of patients underwent a fresh embryo transfer, while 16.6% had at least one FET.

The cumulative clinical pregnancy rate (CCPR) per cycle following oocyte retrieval was determined to be 32.6%, accompanied by a CLBR of 25.5%, resulting in a total of 1,601 live newborns. These rates reflect only the first occurrence of pregnancy and live birth for each couple. A subset of 45 couples experienced two pregnancies, while 2 couples had three pregnancies. Notably, 10 couples achieved two live births.

**Figure 4:**
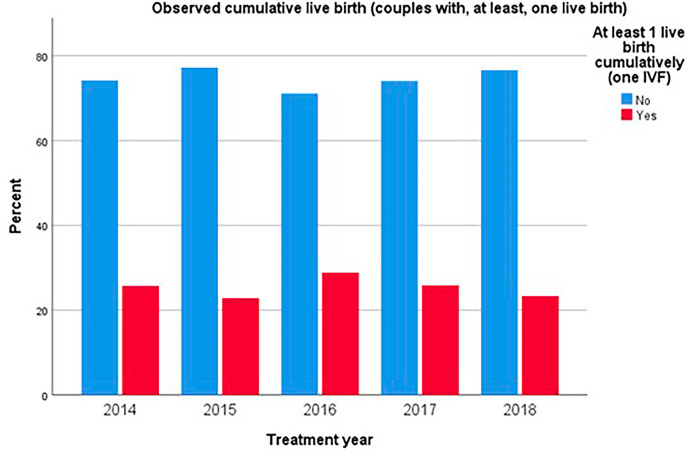
Observed cumulative live birth rate (CLBR) by year of treatment.

The probability of achieving at least one live birth varied across the years, peaking in 2016 at 28.9% and reaching a low of 22.8% in 2015 (*p*=0.11; Figure 4). This trend corresponds with the probability of at least one clinical pregnancy, which was highest in 2016 at 35.4% and lowest in 2015 and 2018, both at 30% (*p*=0.028).

#### Oocyte number

**Figure 5:**
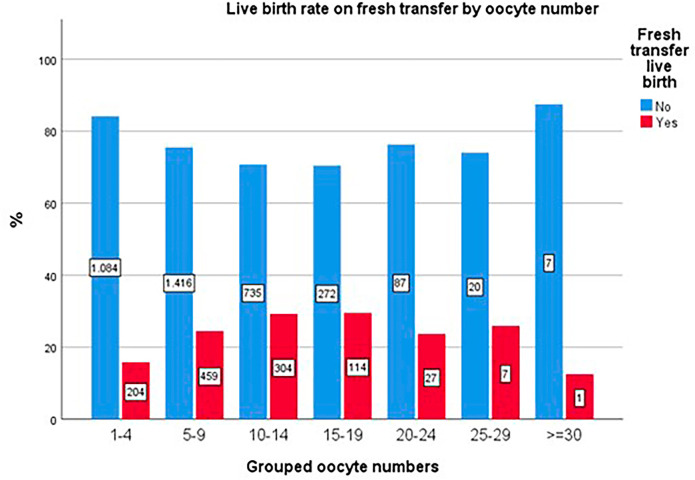
Live birth rate on fresh embryo transfer by number of oocytes.

The live birth rate associated with fresh transfer cycles exhibited a significant decline following a plateau at approximately 15 retrieved oocytes (*p*<0.001; Figure 5).

**Figure 6:**
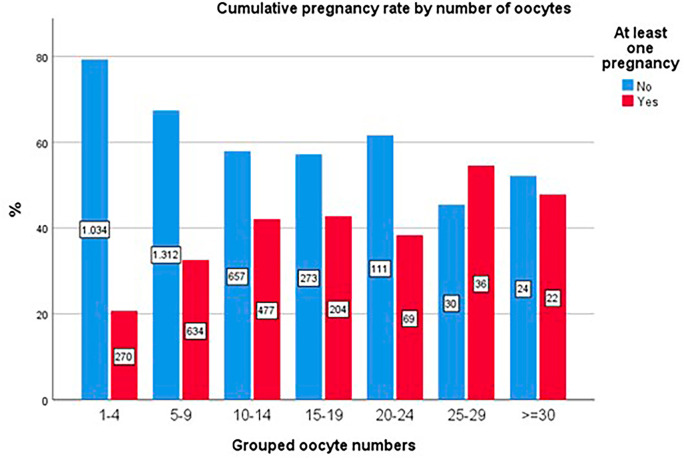
Cumulative clinical pregnancy rates (CCPR) by number of oocytes.

**Figure 7:**
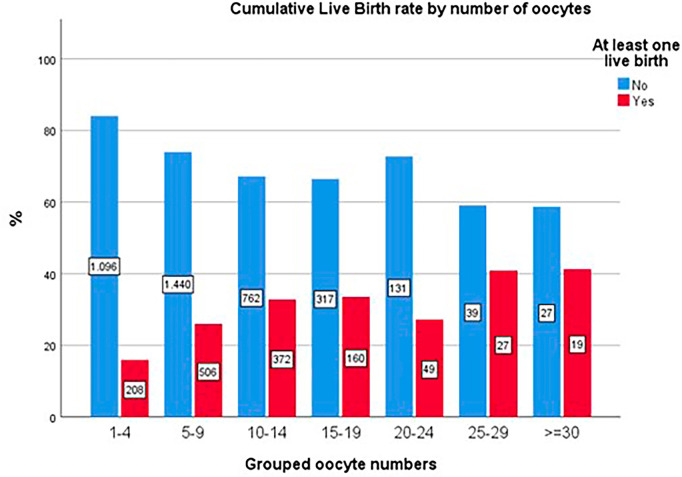
Cumulative live birth rates (CLBR) by number of oocytes.

In contrast, the cumulative pregnancy rate and live birth rate per cycle demonstrated no such inflection, with cumulative rates continuing to increase across the entire range of oocyte collection (*p*<0.001; Figures 6 and 7).

**Figure 8:**
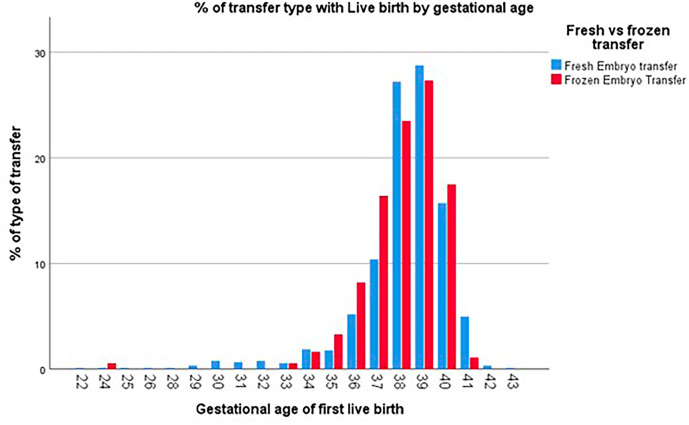
Percentage of gestational age at live birth by fresh/frozen embryo transfer (FET) in singletons.

Additionally, no statistically significant differences were identified concerning prematurity or gestational age at birth when comparing singleton live births resulting from frozen and fresh transfer cycles (Figure 8).

### Regression Model

A logistic regression model was developed to assess the probability of achieving at least one live birth, incorporating variables identified in the literature as associated with the success of IVF cycles (Dhillon *et al*., 2016). The model underwent iterative optimization for stability and relevance, as detailed in Table 10.

**Table 10:** Regression Model [*Model -2 Log likelihood: 5742.530*].

	B	S.E.	Wald	df	Sig.	Exp(B)	95% C.I. for EXP(B)	
							Lower	Upper
Female is obese	-.287	.117	5.991	1	**.014**	.751	.597	.944
Female age (per year)	-.115	.016	53.263	1	**.000**	.891	.864	.919
Infertility duration (per month)	-.003	.001	4.394	1	**.036**	.997	.995	1.000
Oocyte number at Oocyte Pick Up	-.133	.046	8.448	1	**.004**	.876	.801	.958
Female age by Oocyte number	.005	.001	15.536	1	**.000**	1.005	1.003	1.008
Unexplained Infertility diagnosis	-.174	.082	4.513	1	**.034**	.840	.716	.987
Is smoker or previous smoker	-.105	.081	1.668	1	.197	.901	.768	1.056
Constant	2.637	.545	23.444	1	**.000**	13.976		

The regression equation is expressed as follows:

*logOR*= 2.637 -0,287*(obese(1))-0,115*(age in years)-0,003*(inf duration in months)+0,005*(age*oocyte number)-0,174*(unexpl. Infert(1))-0,105*(smoker or previous smoker(1))

Using this model, an individual who is 27 years old, of normal weight, a non-smoker, with 10 oocytes retrieved, a duration of infertility of 20 months, and no unexplained infertility factor would have an estimated probability of live birth ( $\frac{\text{OR}}{\text{OR}+1}$ ) of approximately 38%. Conversely, a 37-year-old individual who is a smoker, obese, has unexplained infertility, and the same retrieval of 10 oocytes over 20 months would demonstrate a significantly lower probability of live birth at approximately 15%.

## DISCUSSION

The rise in the incidence of IVF treatments necessitates enhanced rigor in monitoring these procedures. The systematic implementation of organized databases facilitates optimized data collection, thereby advancing knowledge and surveillance of the treatments administered. Underreporting of adverse events may contribute to an overestimation of the safety of these interventions, as indicated by the latest findings from the European IVF Monitoring Consortium (EIM), underscoring the importance of systematic data collection (De Geyter *et al*., 2020). Such data will enable a deeper understanding of national performance outcomes in IVF.

## LIMITATIONS AND STRENGTHS

This study encompasses several limitations. It is a retrospective analysis contingent upon the accuracy of the medical records from individual centers. Although a high degree of consistency was achieved in data extraction, issues remain, including missing data and incorrect classifications. It is plausible that some couples may have previously undergone IVF treatment at other public or private centers; however, this potential overlap is unlikely to significantly affect the findings. The male factor had a significant statistical impact, nevertheless, the limitations of this study include the absence of detailed information on specific male factors in the Portuguese national ART registry platform. This multicenter study includes all public centers in Northern and Central Portugal, making it, to the best of current knowledge, the largest investigation to date involving infertile couples undergoing IVF in Portugal, and the only one to calculate cumulative outcomes relative to this population.

### Population

Of particular significance within this cohort of ART treatments is the steady increase in the age of women undergoing their first IVF treatment over the years. This trend aligns with the increasing average age of women at the birth of their first child, as documented in the literature (Matthews & Hamilton, 2009). Such an increase may raise the probability of infertility and delay diagnosis, subsequently diminishing the chances of successful ART outcomes. Female age remains the principal determinant of IVF success, both in the published literature (Adebayo *et al*., 2023) and in current data. Notably, analysis shows a statistically significant increase in female age across the examined periods (2014-2016/2017/2018; 2015-2017/2018; 2016-2018). Additionally, a statistically significant rise in the proportion of women over 38 years old and a decrease in the proportion of women under 31 years old were observed.

Among the population studied, a high prevalence of overweight and obesity was noted, with rates of 33.8% for females and 61.5% for males. Female obesity has been associated with a negative independent impact on cumulative IVF success (Sermondade *et al*., 2019), as demonstrated in logistic regression analysis. The predominant causes of infertility identified were male factors, both in isolation and in conjunction with female factors (31.3% and 51.9%, respectively), followed by female-related causes, particularly ovulatory and anatomical factors. The prevalence of endometriosis (5.4%) may be underestimated, as broad diagnostic categories could lead to misclassification; endometriosis may co-occur with other male or female factors that might have been classified as mixed causes.

In our study, male factor infertility was identified as a significant contributor to the overall infertility experienced by couples when combined with female factors, which highlights the need for targeted interventions that address these prevalent conditions. Future research should focus on understanding the underlying causes of male infertility, as these insights could lead to improved counseling and treatment strategies (Brincat *et al*., 2015). Moreover, this aligns with some studies that suggest that addressing male factors in the ART process could enhance overall success rates (Simon *et al*., 2014; Jimbo *et al*., 2022).

### IVF Cycles

Despite the effectiveness of both GnRH agonist and antagonist protocols in suppressing premature luteinizing hormone (LH) surges through reversible blockade of pituitary gonadotropin secretion, the precise impact of these protocols on clinical outcomes in IVF and embryo transfer (IVF-ET) remains a topic of debate (Lai *et al*., 2013). Nevertheless, GnRH antagonists are clearly associated with reduced rates of OHSS (Pundir *et al*., 2012). Current findings also reflect a global trend towards the increased utilization of short antagonist protocols in ART cycles without adversely affecting pregnancy rates (Siristatidis *et al*., 2025). Statistical analysis revealed significant annual variations in the use of agonist protocols; however, no clear trend emerged as usage declined steadily from 2015 to 2017 before rising again in 2018. This fluctuation may be attributable to the growing preference for agonist protocols among patients with low ovarian reserve, where the aforementioned OHSS risk is less pertinent. The participating centers exhibited minimal application of natural cycles, consistent with existing literature (Siristatidis *et al.*, 2025).

Half of the couples undergoing their first IVF cycle were able to retrieve six or more mature oocytes and produce three or more embryos. However, only 26% of the total cohort had frozen embryos, although this figure is on the rise, with one-third of couples in 2018 having at least one cryopreserved embryo, and 12.5% resulting from freeze-all cycles. The overall use of ICSI exhibited a slight decline.

Reported complications appear to be underestimated, with a rate of only 0.4%. Although the implementation of strategies such as “freeze-all” may lead to lower incidence rates of complications, including OHSS-which is notably underreported-especially given that 12.5% of cryopreserved embryos originated from freeze-all cycles.

### Fresh Embryo Transfer Cycles

Over two-thirds of couples (71.8%) underwent fresh transfers during their first IVF cycle. The remaining cases, which did not include embryo transfers, comprised instances where no viable embryos were available or cases designated as freeze-all. The observed high rate of cesarean deliveries (48.2%) in fresh cycles may be attributed to the elevated incidence of multiple gestations and the associated obstetric risks.

### Frozen Embryo Transfer (FET) Cycles

In terms of FET cycles, nearly a quarter (23.7%) of couples following an IVF/ICSI cycle initiated at least one FET cycle. This notable rate indicates improvement in cryopreservation technologies and represents an additional opportunity for couples to achieve pregnancy, thereby enhancing cumulative probabilities of successful live birth outcomes. Despite higher LBR and lower miscarriage rates favoring natural cycles, the differences were not statistically significant, consistent with existing literature (Lathi *et al.*, 2015). The live birth rate per transfer was recorded at 21.1%, which, while commendable, indicates potential for further enhancement, given a multiple birth rate of 16.1% that exceeded expectations. It is anticipated that the trend towards eSET may contribute to a reduction in multiple births in both current and future contexts.

As observed in fresh transfer cycles, the cesarean delivery rate among FET was also high at 58%, attributable to the increased prevalence of multiple births and their associated risks. Unfortunately, data regarding mode of labor (spontaneous versus induced) were not available, which would provide further insights into these outcomes. The obstetric outcomes surrounding prematurity, especially in multiple pregnancies, warrant concern. While neonatal outcome data were lacking, the high rates of prematurity noted (also in fresh transfer cycles) and their associated complications necessitate careful consideration of the number of embryos transferred.

### Cumulative Results

Approximately 26% of couples entering a public IVF program achieved a live birth following their first treatment. This outcome is likely influenced by couple-specific characteristics, and the denominator used includes couples lacking oocytes or transferable embryos. It is plausible that the live birth rate is underestimated as some FET cycles were not appropriately matched to their corresponding IVF cycles (leading to exclusion from the dataset), while others are still in progress or have yet to yield results at the time of data collection. Such factors may also partially account for 2018 being one of the years with the lowest CLBR. The selection of a denominator that includes couples without oocytes or embryos reflects a more accurate assessment of results.

The data corroborate findings from the often-cited study by Sunkara *et al*. (2011), which associates oocyte quantity with success in fresh transfers, indicating that retrievals exceeding approximately 15 oocytes may correlate with poorer outcomes (Sunkara *et al*., 2011). Nonetheless, CCPR and CLBR exhibited continued improvement with increased oocyte retrieval, despite the potential for compromised outcomes in fresh transfers within groups achieving higher retrievals. This observation may elucidate the notable dip in the CLBR depicted in the chart for the 20-24 oocyte retrieval group, which experienced fresh transfer attempts in over 120 cycles.

The regression analysis established a robust association between demographic factors and CLBR, confirming that female age is the most significant predictor of ART success, demonstrating an inverse relationship with pregnancy and live birth rates across cycles. The only variable in the model lacking statistical significance was “smoking,” which exhibited inconsistent behavior in the analysis, suggesting either inadequate data collection or high heterogeneity. The binary categorization of smoking status (smoker, former smoker, non-smoker) fails to adequately capture its impact on fertility. Nevertheless, its inclusion remains warranted given its documented significance in prior studies and its biological plausibility (Marston *et al*., 2014). A similar issue pertains to the categorization of infertility diagnoses, which presents challenges due to inherent heterogeneity within groups-except for those classified as having “no factors identified.”

Logistic regression models provide a fundamental understanding of the probability of success expected for specific couples undergoing their first treatment cycle. Given the diverse nature of infertility, these insights should assist in counseling couples and managing expectations while remaining cognizant of the variability inherent in IVF outcomes. The dataset presents instances such as a live birth resulting from the retrieval of two oocytes from a 39-year-old woman, alongside cases where six 30-year-old women, each having retrieved 15 oocytes, necessitated a second IVF cycle. Despite the low occurrence of these “outlier” percentages, it is crucial to recognize the influence of stochastic variability in these outcomes.

## CONCLUSION

This inaugural collaborative project aims to inspire additional colleagues and centers to consolidate their efforts towards the understanding and enhancement of ART in Portugal.

While the data presented herein is valuable, the significance of collaboration in prospective projects and clinical trials may prove to be even greater.
